# Soluble MICA and a *MICA* Variation as Possible Prognostic Biomarkers for HBV-Induced Hepatocellular Carcinoma

**DOI:** 10.1371/journal.pone.0044743

**Published:** 2012-09-14

**Authors:** Vinod Kumar, Paulisally Hau Yi Lo, Hiromi Sawai, Naoya Kato, Atsushi Takahashi, Zhenzhong Deng, Yuji Urabe, Hamdi Mbarek, Katsushi Tokunaga, Yasuhito Tanaka, Masaya Sugiyama, Masashi Mizokami, Ryosuke Muroyama, Ryosuke Tateishi, Masao Omata, Kazuhiko Koike, Chizu Tanikawa, Naoyuki Kamatani, Michiaki Kubo, Yusuke Nakamura, Koichi Matsuda

**Affiliations:** 1 Laboratory of Molecular Medicine, Human Genome Center, Institute of Medical Science, The University of Tokyo, Tokyo, Japan; 2 Center for Genomic Medicine, The Institute of Physical and Chemical Research (RIKEN), Kanagawa, Japan; 3 Department of Human Genetics, Graduate School of Medicine, The University of Tokyo, Tokyo, Japan; 4 Unit of Disease Control Genome Medicine, The Institute of Medical Science, The University of Tokyo, Tokyo, Japan; 5 Department of Clinical Molecular Informative Medicine, Nagoya City University Graduate School of Medical Sciences, Aichi, Japan; 6 The Research Center for Hepatitis and Immunology, National Center for Global Health and Medicine, Chiba, Japan; 7 Department of Gastroenterology, Graduate School of Medicine, The University of Tokyo, Tokyo, Japan; University of Modena & Reggio Emilia, Italy

## Abstract

MHC class I polypeptide-related chain A (MICA) molecule is induced in response to viral infection and various types of stress. We recently reported that a single nucleotide polymorphism (SNP) rs2596542 located in the *MICA* promoter region was significantly associated with the risk for hepatitis C virus (HCV)-induced hepatocellular carcinoma (HCC) and also with serum levels of soluble MICA (sMICA). In this study, we focused on the possible involvement of MICA in liver carcinogenesis related to hepatitis B virus (HBV) infection and examined correlation between the *MICA* polymorphism and the serum sMICA levels in HBV-induced HCC patients. The genetic association analysis revealed a nominal association with an SNP rs2596542; a G allele was considered to increase the risk of HBV-induced HCC (P = 0.029 with odds ratio of 1.19). We also found a significant elevation of sMICA in HBV-induced HCC cases. Moreover, a G allele of SNP rs2596542 was significantly associated with increased sMICA levels (P = 0.009). Interestingly, HCC patients with the high serum level of sMICA (>5 pg/ml) exhibited poorer prognosis than those with the low serum level of sMICA (≤5 pg/ml) (P = 0.008). Thus, our results highlight the importance of *MICA* genetic variations and the significance of sMICA as a predictive biomarker for HBV-induced HCC.

## Introduction

Hepatocellular carcinoma (HCC) reveals a very high mortality rate that is ranked the third among all cancers in the world [1]. HCC is known to develop in a multistep process which has been related to various risk factors such as genetic factors, environment toxins, alcohol and drug abuse, autoimmune disorders, elevated hepatic iron levels, obesity, and hepatotropic viral infections [2]. Among them, chronic infection with hepatitis B virus (HBV) is one of the major etiological factors for developing HCC with considerable regional variations ranging from 20% of HCC cases in Japan to 65% in China [3].

Interestingly, clinical outcome after the exposure to HBV considerably varies between individuals. The great majority of individuals infected with HBV spontaneously eliminate the viruses, but a subset of patients show the persistent chronic hepatitis B infection (CHB), and then progresses to liver cirrhosis and HCC through a complex interplay between multiple genetic and environmental factors [4]. In this regard, genome wide association studies (GWAS) using single nucleotide polymorphisms (SNPs) have highlighted the importance of genetic factors in the pathogenesis of various diseases including CHB as well as HBV-induced HCC [5], [6], [7], [8], [9], [10], [11], [12], [13]. Recently, we identified a genetic variant located at 4.7 kb upstream of the *MHC class I polypeptide-related chain A* (*MICA*) gene to be strongly associated with hepatitis C virus (HCV) -induced HCC development [14].

MICA is highly expressed on viral-infected cells or cancer cells, and acts as ligand for NKG2D to activate antitumor effects of Natural killer (NK) cells and CD8^+^ T cells [15], [16]. Our previous results indicated that a G allele of SNP rs2596542 was significantly associated with the lower cancer risk and the higher level of soluble MICA (sMICA) in the serum of HCV-induced HCC patients, demonstrating the possible role of MICA as a tumor suppressor. However, elevation of serum sMICA was shown to be associated with poor prognosis in various cancer patients [17], [18], [19], [20]. Matrix metalloproteinases (MMPs) can cleave MICA at a transmembrane domain [21] and release sMICA proteins from cells. Since sMICA was shown to inhibit the antitumor effects of NK cells and CD8^+^ T cells by reduction of their affinity to binding to target cells [22], [23], the effect of MICA in cancer cells would be modulated by the expression of MMPs. To elucidate the role of MICA in HBV-induced hepatocellular carcinogenesis, we here report analysis of the *MICA* polymorphism and serum sMICA level in HBV-induced HCC cases.

## Materials and Methods

### Study participants

The demographic details of study participants are summarized in Table 1. A total of 181 HCC cases, 597 CHB patients, and 4,549 non-HBV controls were obtained from BioBank Japan that was initiated in 2003 with the funding from the Ministry of Education, Culture, Sports, Science and Technology, Japan [24]. In the Biobank Japan Project, DNA and serum of patients with 47 diseases were collected through collaborating network of 66 hospitals throughout Japan. List of participating hospitals is shown in the following website (http://biobankjp.org/plan/member_hospital.html). A total of 226 HCC cases, 102 CHB patients, and 174 healthy controls were additionally obtained from the University of Tokyo. The diagnosis of chronic hepatitis B was conducted on the basis of HBsAg-seropositivity and elevated serum aminotransferase levels for more than six months according to the guideline for diagnosis and treatment of chronic hepatitis (The Japan Society of Hepatology, http://www.jsh.or.jp/medical/gudelines/index.html). Control Japanese DNA samples (n = 934) were obtained from Osaka-Midosuji Rotary Club, Osaka, Japan. All HCC patients were histopathologically diagnosed. Overall survival was defined as the time from blood sampling for sMICA test to the date of death due to HCC. Patients who were alive on the date of last follow-up were censored on that date. All participants provided written informed consent. This research project was approved by the ethics committee of the University of Tokyo and the ethics committee of RIKEN. All clinical assessments and specimen collections were conducted according to Declaration of Helsinki principles.

**Table 1:** Demographic details of subjects analyzed.

Subjects	Source	Genotyping platform	Number of Sample	Female (%)	Age (mean+/−sd)
Liver Cancer	BioBank Japan	Illumina Human Hap610-Quad	181	17.9	62.94±9.42
	University of Tokyo	Invader assay	226		
Control	BioBank Japan	Illumina Human Hap550v3	4549	47.95	55.19±12.5
	Osaka**	Illumina Human Hap550v3	934		
	University of Tokyo	Invader assay	174		
Chronic hepatitis B*	BioBank Japan	Invader assay	597	45.66	61.31±12.6
	University of Tokyo	Invader assay	102		

* Chronic hepatitis B patients without liver cirrhosis and liver cancer during enrollment.

** Healthy volunteers from Osaka Midosuji Rotary Club, Osaka, Japan.

### SNP genotyping

Genotyping platforms used in this study were shown in Table 1. We genotyped 181 HCC cases and 5,483 non-HBV control samples using either Illumina Human Hap610-Quad or Human Hap550v3. The other samples were genotyped at SNP rs2596542 by the Invader assay system (Third Wave Technologies, Madison, WI).

### *MICA* variable number tandem repeat (VNTR) locus genotyping

Genotyping of the *MICA* VNTR locus in 176 HBV-induced HCC samples was performed using the primers reported previously by the method recommended by Applied Biosystems (Foster City, CA) [14]. Briefly, the 5′ end of forward primer was labeled with 6-FAM, and reverse primer was modified with GTGTCTT non-random sequence at the 5′ end to promote Plus A addition. The PCR products were mixed with Hi-Di Formamide and GeneScan-600 LIZ size standard, and separated by GeneScan system on a 3730×l DNA analyzer (Applied Biosystems, Foster City, CA). GeneMapper software (Applied Biosystems, Foster City, CA) was employed to assign the repeat fragment size (Figure S1).

### Quantification of soluble MICA

We obtained serum samples of 111 HBV-positive HCC samples, 129 HCV-positive HCC samples, and 60 non-HBV controls from Biobank Japan. Soluble MICA levels were measured by sandwich enzyme-linked immunosorbent assay, as described in the manufacturer's instructions (R&D Systems, Minneapolis, MN).

### Statistical analysis

The association between an SNP rs2596542 and HBV-induced HCC was tested by Cochran-Armitage trend test. The Odds ratios were calculated by considering a major allele as a reference. Statistical comparisons between genotypes and sMICA levels were performed by Kruskal-Wallis test (if more than two classes for comparison) or Wilcoxon rank test using R. Overall survival rate of the patients was analyzed by Kaplan-Meier method in combination with log-rank test with SPSS 20 software. The period for the survival analysis was calculated from the date of blood sampling to the recorded date of death or the last follow-up date. Differences with a P value of <0.05 were considered statistically significant.

## Results

### Association of SNP rs2596542 with HBV-induced HCC

In order to examine the effect of rs2596542 genotypes on the susceptibility to HBV-induced HCC, a total of 407 HCC cases and 5,657 healthy controls were genotyped. The Cochran Armitage trend test of the data revealed a nominal association between HBV-induced HCC and rs2596542 in which a risk allele G was more frequent among HBV-induced HCC cases than an A allele (P = 0.029, OR = 1.19, 95% CI: 1.02–1.4; Table 2). To further investigate the effect of rs2596542 on the progression from CHB to HBV-induced HCC, we genotyped a total of 699 CHB cases without HCC. Although the progression risk from CHB to HBV-induced HCC was not statistically significant with rs2596542 (P = 0.197 by the Cochran Armitage trend test with an allelic OR = 1.3 (0.94–1.36); Table 2), we found a similar trend of association in which the frequency of a risk-allele G was higher among HBV-induced HCC patients than that of CHB subjects. Since we previously revealed that an A allele was associated with a higher risk of HCV-induced HCC with OR of 1.36 [14], the s2596542 alleles that increased the risk of HCC were opposite in HBV-induced HCC and HCV-induced HCC.

**Table 2:** Association between HCC and rs2596542.

SNP	Comparison	Chr	Locus	Case MAF	Control MAF	*P* *	OR*	95% CI
rs2596542	HCC vs. Healthy control	6	*MICA*	0.294	0.332	0.029	1.19	1.02–1.4
rs2596542	HCC vs. CHB	6	*MICA*	0.294	0.320	0.197	1.13	0.94–1.36

Note: 407 HCC cases, 699 CHB subjects and 5,657 non-HBV controls were used in the analysis.

Chr.,chromosome; MAF, minor allele frequency; OR, odds ratio for minor allele; CI, confidence interval.

* Obtained by Armitage trend test.

### Soluble MICA levels are associated with SNP rs2596542

**Figure 1:**
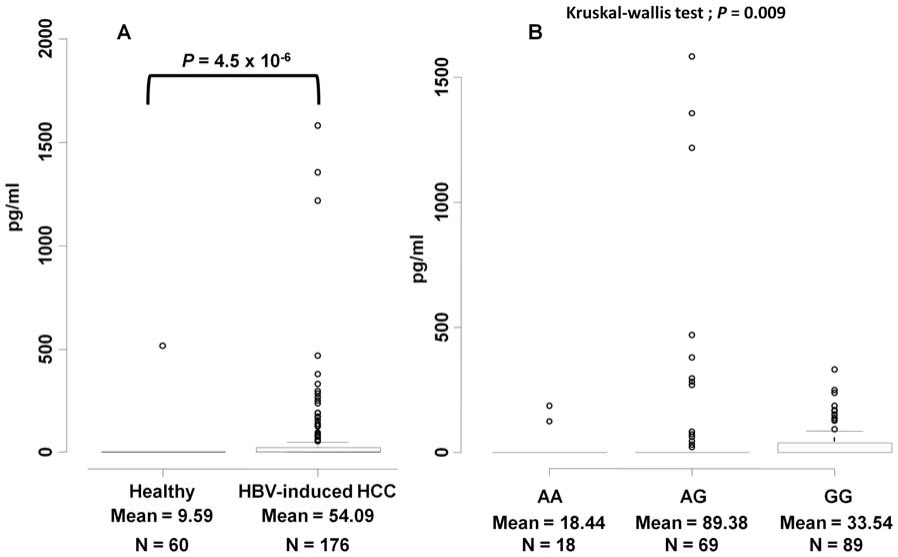
Soluble MICA levels are associated with HBV-related HCC. (A) Correlation between soluble MICA levels and HBV-induced HCC subjects. The y-axis displays the concentration of soluble MICA in pg/ml. The number of independent samples tested in each group is shown in the x-axis. Each group is shown as a box plot and the mean values are shown in the x-axis. The difference between two groups is tested by Wilcoxon rank test. The box plots are plotted using default settings in R. (B) Correlation between soluble MICA levels and rs2596542 genotype in HBV-positive HCC subjects. The x-axis shows the genotypes at rs2596542 and y-axis display the concentration of soluble MICA in pg/ml. Each group is shown as a box plot. *P* = 0.027 and 0.013 for AA vs. GG and AA vs. AG, respectively. The association between genotypes and sMICA levels was tested by Kruskal-wallis test, whereas the difference in the sMICA levels between AA and GG is tested by Wilcoxon rank test. The box plots are plotted using default settings in R.

We subsequently performed measurement of soluble MICA (sMICA) in serum samples using the ELISA method in 176 HBV-positive HCC cases and 60 non-HBV controls. Nearly 30% of the HBV-induced HCC cases revealed the serum sMICA level of >5 pg/ml (defined as high) while the all control individuals except one showed that of ≤5 pg/ml (defined as low) (P = 4.5×10^−6^; Figure 1A). Then, we examined correlation between SNP rs2596542 genotypes and serum sMICA levels in HBV-positive HCC cases. Interestingly, rs2596542 genotypes were significantly associated with serum sMICA levels (P = 0.009; Figure 1B); 39% of individuals with the GG genotype and 20% of those with the AG genotype were classified as high for serum sMICA, but only 11% of those with the AA genotype were classified as high (AA+AG vs GG; P = 0.003) (Figure 1B). These findings were similar with our previous reports in which a G allele was associated with higher serum sMICA levels in HCV-induced HCC patients [14].

### Negative association of variable number of tandem repeat (VNTR) with sMICA level

The *MICA* gene harbors a VNTR locus in exon 5 that consists of 4, 5, 6, or 9 repeats of GCT as well as a G nucleotide insertion into a five-repeat allele (referred as A4, A5, A6, A9, and A5.1, respectively). The insertion of G (A5.1) causes a premature translation termination and results in loss of a transmembrane domain, which may produce the shorter form of the MICA protein that is likely be secreted into serum [25]. However, the association of this VNTR locus with serum sMICA level was controversial among studies [14], [26], [27], [28]. Therefore, we examined the association between the VNTR locus and sMICA level in HBV-induced HCC patients, and found no significant association (Figure S1 and S2), concordant with our previous report for HCV-induced HCC patients [14].

### Soluble MICA levels are associated with survival of HCC patients

**Figure 2:**
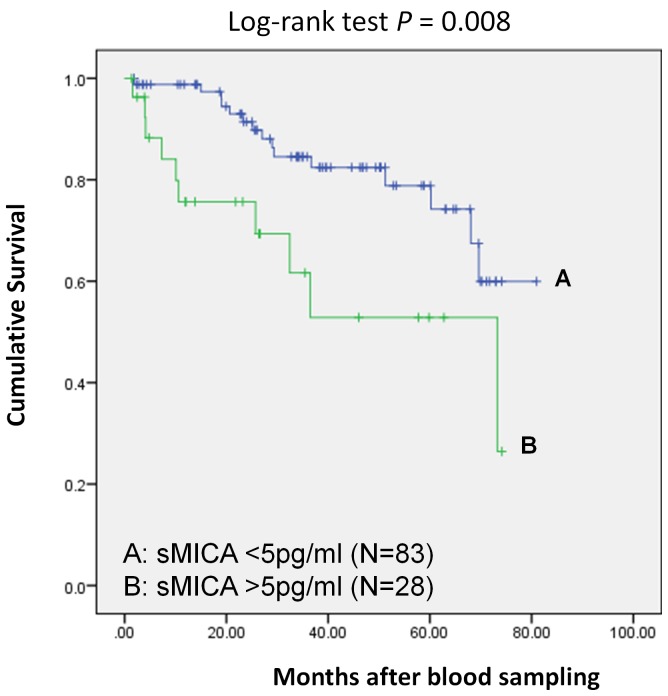
Kaplan-Meier curves of the patients with HBV-induced HCC. The patients were divided into two groups according to their sMICA concentration (high: >5 pg/ml and low:  = <5 pg/ml). Statistical difference was analyzed by log-rank test. The y-axis shows the cumulative survival probability and x-axis display the months of the patient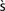 survival after blood sampling.

In order to evaluate the prognostic significance of serum sMICA levels in HCC patients, we performed survival analysis of HCC patients. A total of 111 HBV-infected HCC patients and 129 HCV-infected HCC patients were included in this analysis. The mean survival period for HBV- and HCV-infected patients with less than 5 pg/ml of serum sMICA were 67.1 months (95% CI: 61.1–73.1, n = 83), and 58.2 months (95% CI: 51.4–65.0, n = 85), respectively. On the other hand, for patients with more than 5 pg/ml of serum sMICA, the mean survival periods were 47.8 months (95% CI: 34.8–30.9, n = 28) for HBV-induced HCC patients and 59.5 months (95% CI: 51.9–67.1, n = 44) for HCV-induced HCC patients. The Kaplan-Maier analysis and log-rank test indicated that among HBV-induced HCC subjects, the patients in the high serum sMICA group showed a significantly shorter survival than those in the low serum sMICA (P = 0.008; Figure 2). In addition, we performed multi-variate analysis to test whether sMICA is an independent prognostic factor by including age and gender as covariates. The results revealed significant association of sMICA levels with overall survival (P = 0.017) but not with age and gender (Table S1).However, we found no association between the serum sMICA level and the overall survival in the HCV-induced HCC subjects (P = 0.414; Figure S3). Taken together, our findings imply the distinct roles of the *MICA* variation and sMICA between HBV- and HCV-induced hepatocellular carcinogenesis.

### Vascular invasion in HBV-related HCC patients is associated with soluble MICA levels

**Figure 3:**
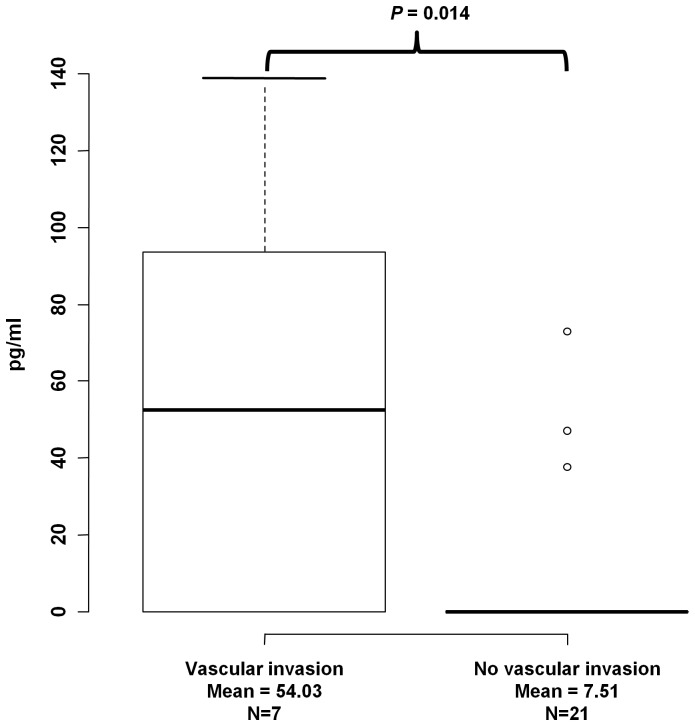
Correlation between soluble MICA levels and vascular invasion in HBV-induced HCC subjects. The y-axis displays the concentration of soluble MICA in pg/ml. The number of independent samples tested in each group is shown in the x-axis. Each group is shown as a box plot and the mean values are shown in the x-axis. The difference between two groups is tested by Wilcoxon rank test. The box plots are plotted using default settings in R.

Since sMICA levels were associated with the overall survival of HBV-related HCC patients, we tested whether sMICA levels affect survival through modulating invasive properties of tumors or size of the tumors. We tested the association between sMICA levels and vascular invasion in 35 HBV-related HCC cases, among whom 7 cases were positive and 21 cases were negative for vascular invasion. We found significant association between sMICA levels and vascular invasion (Figure 3; P = 0.014) in which 7 cases with positive vascular invasion showed high levels of sMICA (mean = 54 pg/ml) than 21 cases without vascular invasion (mean = 7.51 pg/ml). However, we found no association between tumor size and sMICA levels (P = 0.56; data not shown). These results suggest that sMICA may reduce the survival of HBV-related HCC patients by affecting the invasive properties of tumors.

## Discussion

Several mechanisms such as HBV-genome integration into host chromosomal DNA [29] and effects of viral proteins including HBx [30] are shown to contribute to development and progression of HCC, while the immune cells such as NK and T cells function as key antiviral and antitumor effectors. MICA protein has been considered as a stress marker of gastrointestinal epithelial cells because of its induced expression by several external stimuli such as heat, DNA damage, and viral infections [31], [32], [33], [34]. Here, we examined the association of rs2596542 and serum sMICA levels with HBV-induced HCC. Like in HCV-induced HCC [14], our results from ELISA revealed a significantly higher proportion of high serum sMICA cases (nearly 30%) in the HBV-induced HCC group, compared to non-HBV individuals (1.7%). Moreover, the serum sMICA level was significantly associated with rs2596542, but not with the copy number differences of the VNTR locus, as concordant with our previous report [14].

Several studies have already indicated the roles of sMICA as prognostic markers for different types of malignant diseases [17], [18], [19], [20]. Therefore, it is of medical importance to test whether serum sMICA levels can be used as a prognostic marker for patients with HCC. To our best knowledge, this is the first study to demonstrate the prognostic potential of sMICA for HBV-positive HCC patients; we found 19.3 months of improvement in survival among patients carrying less than 5 pg/ml of serum sMICA, compared to those having more than 5 pg/ml.

On the contrary, we found no significant correlation between sMICA levels and the prognosis of HCV-induced HCC cases. These opposite effects of *MICA* variation could be explained by the following mechanism. The individuals who carry the G allele would express high levels of membrane-bound MICA upon HCV infection and thus lead to the activation of immune cells against virus infected cells. On one hand, HBV infection results in increased expression of membrane-bound MICA as well as MMPs through viral protein HBx [35], which would result in the elevation of sMICA and the reduction of membrane-bound MICA. Since sMICA could block CD8+T cells, NK-CTL, and NK cells, higher sMICA would cause the inactivation of immune surveillance system against HBV infected cells. In other words, HBV may use this strategy to evade immune response and hence, higher levels of sMICA could be associated with lower survival rate among HBV-associated HCC. On the other hand, since HCV is not known to induce the cleavage of membrane bound MICA, individuals with low level membrane bound MICA expression (carriers of rs2596542-allele A) could be inherently susceptible for HCV-induced HCC. Thus, HBx-medicated induction of MMPs could partially explain the intriguing contradictory effect of MICA between HBV-induced HCC and HCV-induced HCC. Since we observed significant correlation of sMICA levels with vascular invasion, it may be the case that high levels of sMICA cause poor prognosis of HBV-related HCC cases by making tumors more aggressive and invasive. However it is important in future to determine the ratio of membrane-bound MICA to sMICA in case of HCV- and HBV-related HCC.

Interestingly, the immune therapy against melanoma patients induced the production of auto-antibodies against MICA [36]. Anti-MICA antibodies would exert antitumor effects through antibody-dependent cellular cytotoxicity against cells expressing membrane-bound MICA and/or activation of NK cells by inhibiting the sMICA-NKG2D interaction. However, further studies are necessary, using well-defined HBV-related HCC cohort, to investigate whether sMICA levels could be included as an additional factor to predict the survival rate among HBV-related HCC subjects. Taken together, our results indicate the potential of *MICA* variant and sMICA as prognostic biomarkers. Thus, MICA could be a useful therapeutic target for HBV-induced HCC.

## Supporting Information

Figure S1 **MICA repeat genotyping using capillary-based method.** The alleles are annotated using GeneMapper software based on the size of the PCR product (185 bp = A4 allele, 188 bp = A5, 189 bp = A5.1, 191 bp = A6 and 200 bp = A9). The inset at the base of each peak shows the size of the PCR product with corresponding allele call by the software. The figure display all observed heterozygotes at A5.1 allele.
(TIF)

Figure S2 **MICA VNTR alleles are not associated with soluble MICA levels.** Each group is shown as a box plot. The difference in the sMICA values among each group is tested by Wilcoxon rank test. The box plots are plotted using default settings in R.
(TIF)

Figure S3
**Kaplan-Meier curves of the patients with HCV-induced HCC.** The patients were divided into two groups according to their sMICA concentration (<5 pg/ml or >5 pg/ml). Statistical difference was analyzed by log-rank test. The y-axis shows the cumulative survival probability and x-axis display the months of the patient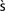 survival after blood sampling.(TIF)

Table S1 Clinical parameters of HBV-related HCC patients available for prognostic analyses.
(XLS)
