# Supplementary figures and images for: Soluble MICA and a MICA Variation as Possible Prognostic Biomarkers for HBV-Induced Hepatocellular Carcinoma
Source: PLoS One. 2012 Sep 14;7(9):e44743. doi: 10.1371/journal.pone.0044743 (PMC3443094; doi:10.1371/journal.pone.0044743)

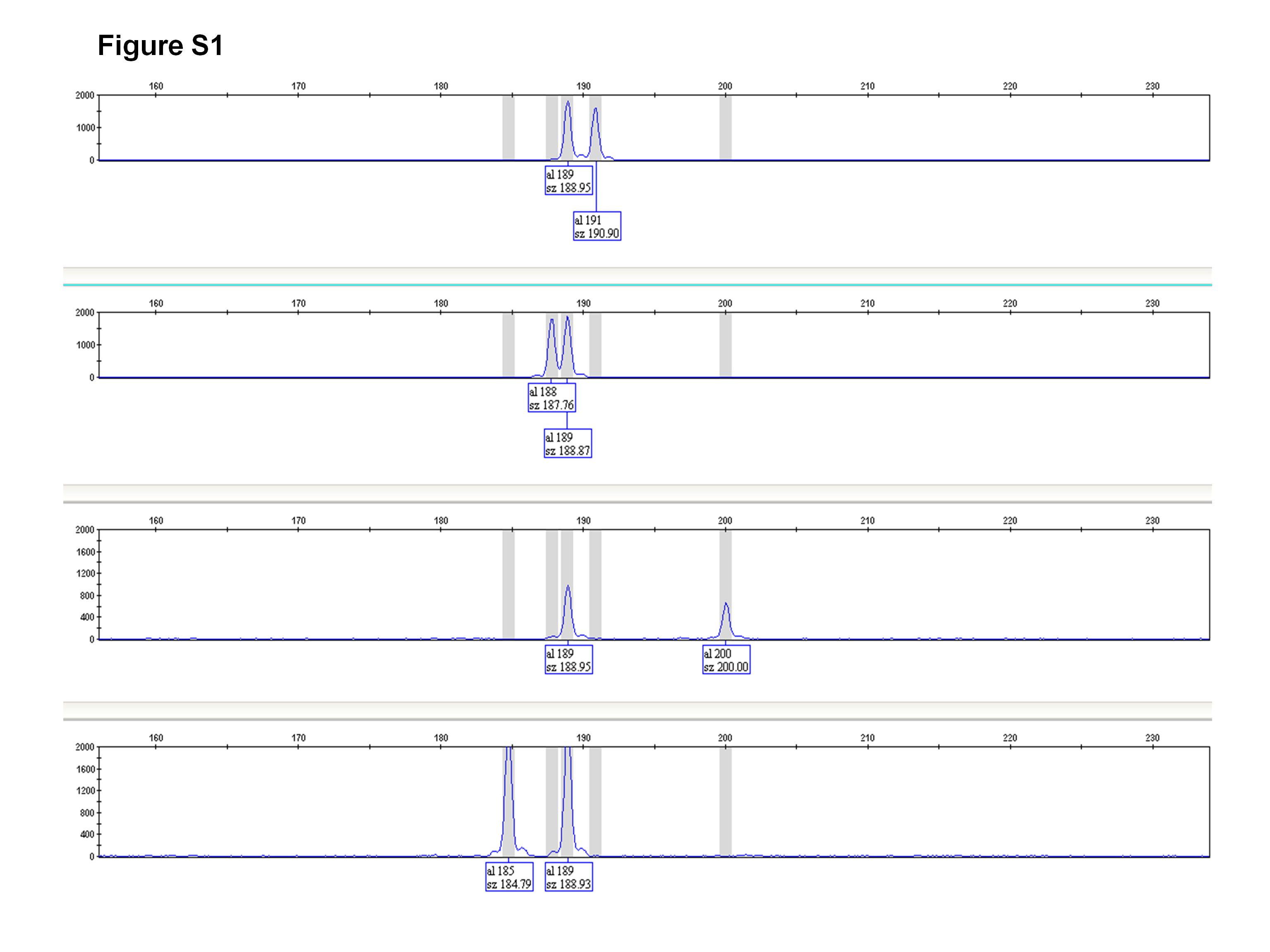

Supplement: Figure S1 — MICA repeat genotyping using capillary-based method. The alleles are annotated using GeneMapper software based on the size of the PCR product (185 bp = A4 allele, 188 bp = A5, 189 bp = A5.1, 191 bp = A6 and 200 bp = A9). The inset at the base of each peak shows the size of the PCR product with corresponding allele call by the software. The figure display all observed heterozygotes at A5.1 allele. (TIF) [file pone.0044743.s001.tif]

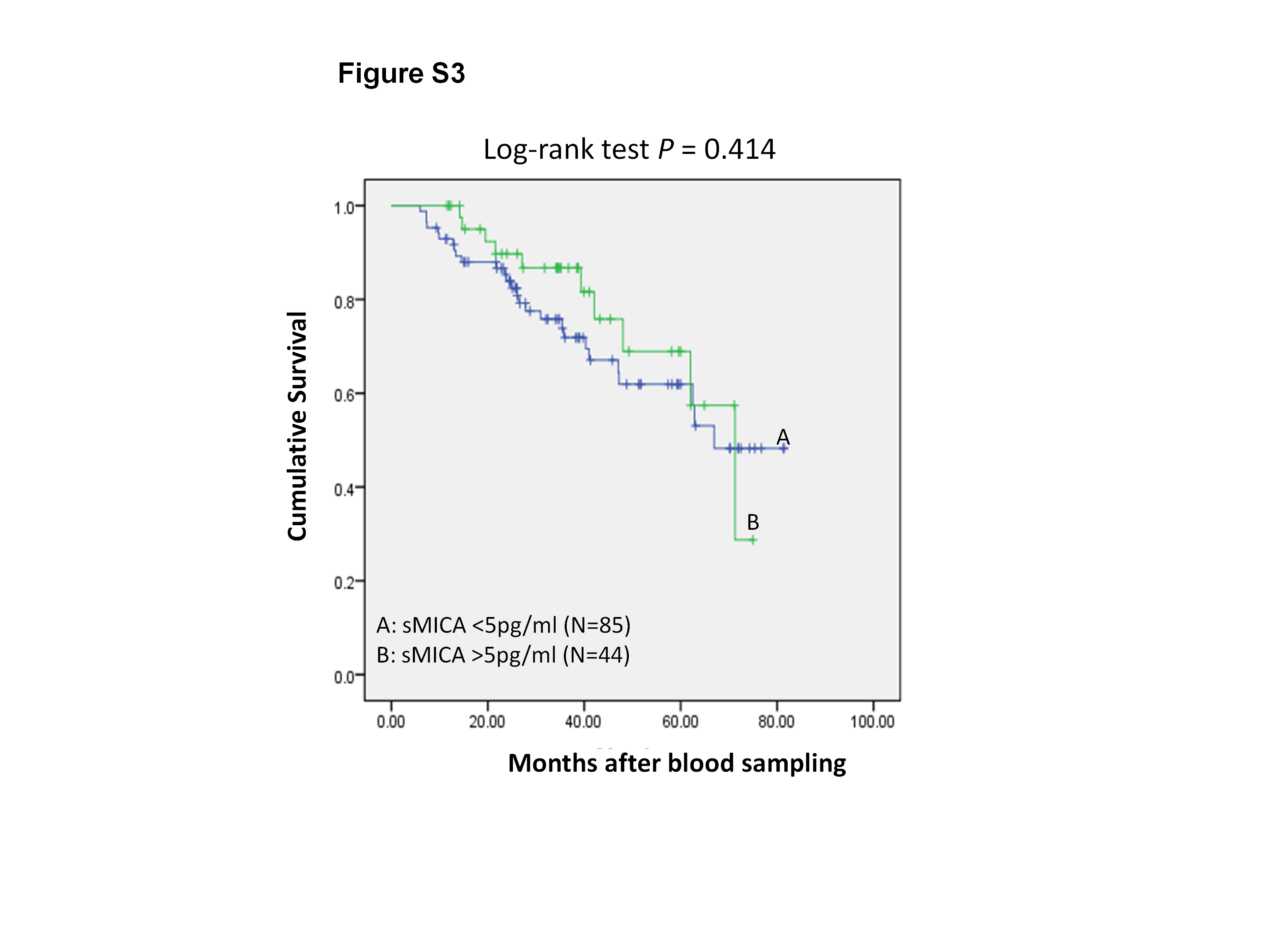

Supplement: Figure S3 — Kaplan-Meier curves of the patients with HCV-induced HCC. The patients were divided into two groups according to their sMICA concentration (<5 pg/ml or >5 pg/ml). Statistical difference was analyzed by log-rank test. The y-axis shows the cumulative survival probability and x-axis display the months of the patient survival after blood sampling. (TIF) [file pone.0044743.s003.tif]
